# The mitochondrial genome of Chinese endemic species *Pseudolimnophila* (*Pseudolimnophila*) *brunneinota* (Diptera: Limoniidae)

**DOI:** 10.1080/23802359.2019.1681313

**Published:** 2019-10-24

**Authors:** Jinlong Ren, Bing Zhang, Ding Yang

**Affiliations:** College of Plant Protection, China Agricultural University, Beijing, China

**Keywords:** Mitochondrial genome, Tipuloidea, phylogenetics, *Pseudolimnophila*

## Abstract

The mitogenome of *Pseudolimnophila* (*Pseudolimnophila*) *brunneinota* was sequenced, the new representative of the subfamily Limnophilinae in mitogenome. The completed mitogenome is 15,985 bp totally, comprising of 13 protein-coding genes, 2 rRNAs, 22 transfer RNAs and control region. All genes have the similar locations and strands with other published species of Tipuloidea. The nucleotide composition biases toward A and T, which together made up 78.00％of the entirety. Bayesian inference analysis strongly supported the monophyly of Chioneinae + Limnophilinae. It suggested that the phylogenetic relationship within Tipuloidea is Pediciidae + (Limoniidae ((Chioneinae + Limnophilinae)+ Limoniinae) + (Tipulidae + Cylindrotomidae)).

## Introduction

Limoniidae is the most taxonomically diverse group in Tipuloidea with 10,666 recognized species in 364 genera and subgenera (Oosterbroek 2019). One of the conflicts in Limoniidae is the monophyly of Limnophilinae (Ribeiro 2008). The mitochondrial (mt) genomes are the excellent molecular marker and have been used widely for phylogenetic studies of insects (Cameron 2014). To date, there are 10 complete or nearly completed Tipuloidea mitochondrial genome sequences in GenBank, The Limnophilinae has 110 genera/subgenera, but only one species (*Paradelphomyia* sp.) has been already sequenced. In order to clarify the phylogeny of Limoniidae, the more limnophilids need to be sequenced. So, we sequenced genus *Pseudolimnophila* Alexander, 1919 (Limnophilinae).

The specimens of *P.* (*P.*) *brunneinota* Alexander, 1933 (Diptera: Limoniidae) (Collecting information: Linzhi, Tibet, China, 29.60°N, 94.36°E, 2938 m. 2017.VII.15. Qicheng Yang) used for this study were stored in the Entomological Museum of China Agricultural University (CAU, specimen accession number: CAURJL20190313). The total genomic DNA was extracted from the whole body (except for head, wings, and hypopygium) of the specimen using the QIAamp DNA Blood Mini Kit (Qiagen, Germany) and stored at −20 °C until needed. The mitogenome was amplified and sequenced as described in our previous study (Wang et al. 2016; Ren et al. 2019). Phylogenetic analysis: the best-fit partitioning scheme and substitution models for partition were determined by PartitionFinder2 (Lanfear et al. 2017). Bayesian inference was analyzed by MrBayes 3.2.6 (Ronquist et al. 2012) under GTR + I+G models and running 10 million generations for dataset (14-taxon sampling, PCG123 + RNA). Genbank accession number of sequenced specimen: *Tipula abdominalis* (N861743.1), *Tipula cockerelliana* (NC_030520), *Nephrotoma tenuipes* (MN053900), *Tipula melanomera gracilispina* (MK864102), *Cylindrotoma* sp. (KT970060.1), *Chionea crassipes gracilistyla* (NC_030519), *Symplecta hybrida* (NC_030519), *Paradelphomyia* sp. (KT970061.1), *Limonia phragmitidis* (MK673118), *Rhipidia chenwenyoungi* (KT970063.1), *Pedicia* sp. (KT970062.1), *Trichocera bimacula* (JN861750.1), *Paracladura trichoptera* (NC_016173) and *Sylvicola fenestralis* (NC_016176). The completed mitogenome of *P. brunneinota* is 15,985 bp. It encoded 13 PCGs, 22 tRNA genes, and 2 rRNA genes and the control region. All genes have similar locations and strands with that of other published Tipuloidea species. The nucleotide composition of the mitogenome was biased toward A and T, with 78.00% of A + T content (A = 39.60%, T = 38.40%, G = 8.90%, C = 13.10%,). The A + T content of PCGs, tRNAs, and rRNAs is 76.50, 79.30, and 81.3%, respectively. The total length of all 13 PCGs of *P. brunneinota* is 12,961 bp. Two PCGs (*ATP8* and *NAD2*) initiated with ATT codons and seven PCGs (*ATP6*, *COX2*, *COX3*, *CYTB*, *NAD4, NAD4L,* and *NAD5*) initiated with ATG codons, *NAD1*, while *NAD1* and *NAD3* initiated with ATA, *COX1* and *NAD6* initiated with TTG and ATC as a start codon, respectively.

**Figure 1.**
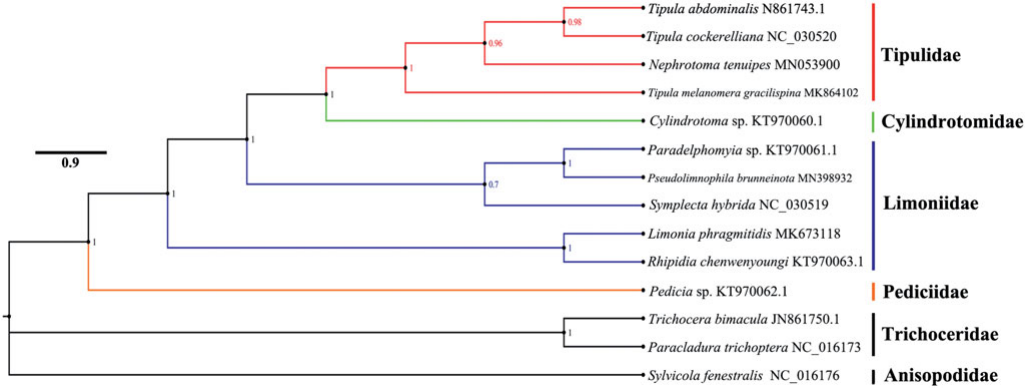
Bayesian analysis (MrBayes) of Tipuloidea phylogeny based on mitochondrial genome. Posterior probability values shown at all nodes.

Bayesian inference (BI) analysis (Mrbayes) was based on the nucleotide sequences of 13 PCGs and 2 rRNAs from 11 species of Tipuloidea (Figure 1). The result showed that the monophyletic Tipuloidea was well supported as the sister group with Trichoceridae + Anisopodidae. Tipulidae is the sister group of Cylindrotomidae and Pediciidae is a basal clade of Tipuloidea. The genera *Pseudolimnophila* and *Paradelphomyia* are sister groups and they combined with *symplecta* to form a monophyletic branch of Limnophilinae + Chioneinae lineage. The phylogenetic relationship within Tipuloidea is very clear with Pediciidae + (Limoniidae ((Chioneinae + Limnophilinae) + Limoniinae) + (Tipulidae + Cylindrotomidae)) (Figure 1). This relationship was supported by the previous studies in part (Petersen et al. 2010; Zhang et al. 2016).
